# UWB and IMU-Based UAV’s Assistance System for Autonomous Landing on a Platform

**DOI:** 10.3390/s22062347

**Published:** 2022-03-18

**Authors:** Aitor Ochoa-de-Eribe-Landaberea, Leticia Zamora-Cadenas, Oier Peñagaricano-Muñoa, Igone Velez

**Affiliations:** 1 CEIT-Basque Research and Technology Alliance (BRTA), Manuel Lardizabal 15, 20018 San Sebastián, Spain; lzamora@ceit.es (L.Z.-C.); ivelez@ceit.es (I.V.); 2 Tecnun School of Engineering, Universidad de Navarra, Manuel Lardizabal 13, 20018 San Sebastián, Spain; 3 Alerion Technologies SL, Paseo de Mikeletegi 73B, Suite 304, 20009 San Sebastián, Spain; oier@aleriontec.com

**Keywords:** UWB, UAV, IMU, data fusion, autonomous landing, RTLS, EKF

## Abstract

This work presents a novel landing assistance system (LAS) capable of locating a drone for a safe landing after its inspection mission. The location of the drone is achieved by a fusion of ultra-wideband (UWB), inertial measurement unit (IMU) and magnetometer data. Unlike other typical landing assistance systems, the UWB fixed sensors are placed around a 2 × 2 m landing platform and two tags are attached to the drone. Since this type of set-up is suboptimal for UWB location systems, a new positioning algorithm is proposed for a correct performance. First, an extended Kalman filter (EKF) algorithm is used to calculate the position of each tag, and then both positions are combined for a more accurate and robust localisation. As a result, the obtained positioning errors can be reduced by 50% compared to a typical UWB-based landing assistance system. Moreover, due to the small demand of space, the proposed landing assistance system can be used almost anywhere and is deployed easily.

## 1. Introduction

The inspection of infrastructures is a necessary task for their correct performance and durability, especially in the case of the energetic, petrochemical, construction or transport sectors. However, sometimes dangerous zones with difficult accessibility must be reached by a human worker (or a group of workers), increasing the risks of the work. For this reason, there is a growing interest in the use of drones or unmanned aerial vehicles (UAVs) for infrastructure inspection [1,2,3,4,5,6]. One of the main advantages of UAVs is their high adaptability to any infrastructure, as they can be used to inspect power transmission lines [1,2,3], surfaces in bridges and roads [4], wind turbines [5] or rail viaduct bearings [6] among others. As a consequence, the infrastructure inspection already makes 45% of the total UAV market [7].

Nevertheless, the use of drones for inspection tasks also has its drawbacks as investment must be made in vehicle and staff training to pilot the UAV. Moreover, since drones must be operated by a person, this solution is still prone to human errors, so the possibility of using autonomous drones should be considered.

The landing manoeuvre is probably one of the riskiest situations of a flight. In the case of an autonomous drone, knowing the real-time location of the vehicle with respect to the landing area is crucial for a successful operation. A positioning error of a few metres could cause significant damage to the drone. A high positioning rate is also important, since adverse conditions such as windy weather could cause sudden velocity changes that could not be detected on time.

In the aeronautic sector it is common to use the global navigation satellite system (GNSS) for an autonomous landing [8]. Nevertheless, this technology is not always available or it is sometimes incapable of giving an acceptable level of accuracy, as can happen when the inside of a tank of a petrochemical plant is to be inspected. For this reason, in the literature, complementary landing assistance systems (LASs) are proposed based on computer vision techniques [9,10,11,12,13,14,15,16,17,18,19], a fusion between computer vision techniques and inertial measurement units (IMUs) [20,21,22,23,24,25], computer vision, IMU and ultrasonic sensors [26], computer vision and a Time-of-Flight-based height sensor [27], computer vision and GNSS [28,29] and even an approach fusing onboard cameras and a robotic total station [30]. The main setback of traditional vision-based systems is their strong dependency on weather or lighting conditions. Coming back to the example of a petrochemical plant, there is no light available inside the tanks, so vision-based systems would fail. Recently, Refs. [12,15] proposed to use convolutional neural networks to detect a marker on the landing area in low illumination environments. However, these networks are yet to be implemented on a resource-limited UAV onboard platform [12,15]. Among the works that use computer vision techniques, Ref. [19] presented really good accuracy. Nevertheless, this work only presented results in short ranges with low velocities of the drone.

Other vision-based approaches use Time-of-Flight (ToF) cameras [31] and a fusion of ToF cameras and ultrasonic sensors [32] for the location of UAVs. However, these proposals install sensors on the ceiling of a room, which can be difficult to do inside the tank of a petrochemical plant.

Recently, millimetre wave radar has been proposed by [33] to detect the presence of UAVs. However, as in the case of [31,32] the UAV detection is undertaken on the ground station. As this information should be sent to the drone for UAV navigation, potential latency problems in the wireless communication link between the drone and ground station could endanger the autonomous landing operation of the UAV.

Other proposals such as [34,35,36] suggest using ultra-wideband (UWB) technology for a safe landing as a passive radar, where similar techniques to those of computer vision are utilised. However, the developed systems are only used to estimate the roughness of the ground where the landing will be performed.

Other recent works such as [37,38,39] have explored the possibility of using active impulse-radio ultra-wideband (IR-UWB) technology to estimate the position of the drone with respect to the landing zone. This technology uses two types of sensors: anchors and tags. Anchors are fixed sensors placed at known locations, and they communicate with the tags to calculate the distances between each anchor and tag. With the measured distances, the position of each tag can be calculated. In order to localise drones for an autonomous landing, Ref. [37] runs simulations to optimise the geometry of the infrastructure formed by UWB anchors and to improve the accuracy of the real-time location system (RTLS). The work of [38] goes a step further and tests the feasibility of a real UWB system to locate UAVs and in [39] a path generation algorithm is proposed for an autonomous landing of the drone. In all of the mentioned works, it is suggested to place the anchors around the landing zone with a separation of tens of metres among them. Such infrastructures are similar to those used in classic RTLSs [40]. However, one of the main drawbacks of UWB-based UAV positioning systems is that it is sometimes impossible or impractical to deploy those large infrastructures. For example, when inspecting off-shore wind turbines, the drone has to land on a boat or a floating platform with little space available. Another case can be the inspection of a tank in a petrochemical plant, where humans are not allowed to stay inside for long time periods for safety reasons, making it impossible to deploy a big infrastructure for a RTLS. A fast and easy deployment is crucial for the latter example.

A possible solution is to use UWB technology with a small infrastructure. In [41], the authors demonstrate that good positioning accuracy can be achieved even if the anchors of a UWB-based AGV (Automated Guided Vehicle) positioning system are not placed in a fixed infrastructure with a big separation between them. Similarly, the authors of [42] proposed to use an anchor infrastructure of around 2 × 2 m to locate a UAV. However, according to [42], the errors of their RTLS are twice as big compared to a deployment where the anchors make up a rectangle of 64 $m^{2}$. Moreover, the experiments were run in a controlled environment, where the authors could easily control all the movements of the drone. In a real environment, wind could cause sudden velocity changes to a UAV. As a consequence, the obtained performance could further decrease because of the limited positioning rate of UWB systems. In fact, the low positioning rate of UWB-based UAV positioning systems poses a big limitation in the positioning accuracy of the system.

There are different methods to improve the positioning accuracy of UWB-based UAV positioning systems. For example, in [43] a particle filter algorithm is proposed for an enhanced performance of UWB for the localisation of drones. However, approaches fusing data from different sensors are more popular. It is very common to fuse UWB data with inertial measurement units, as suggested by [44,45,46]. A third sensor can also be added to the UWB/IMU approach, such as a light scanner in [47], a frequency modulated continuous wave (FMCW) radar in [48] or a real-time kinematic global positioning system (RTK-GPS) in [49]. Another popular approach is to add visual data to the UWB-based RTLS as in [50,51,52,53]. Laser imaging detection and ranging (LIDAR) sensors have also been used to improve the UWB accuracy for UAV location in [54], where a drone had to fly close to a wall. Despite the improved performance of the RTLS proposed in the mentioned works, only one of them uses a simple infrastructure [53], where four UWB anchors are placed around a 1.5 × 1 m pad with a system of visual fiducial tags. The UWB data are fused with the visual and inertial data, resulting in a safe landing. However, it is not known how this system would perform in a dark environment, since the pad must remain in the field of view of the camera.

This paper proposes a novel LAS for autonomous drones that combines data from UWB, IMUs and magnetometers to estimate the position of the drone when approaching or moving away from the landing platform. In this LAS, as in the case of [42], UWB anchors are placed around a small landing platform and two tags are placed on the drone. However, in our case, both tags also have IMUs and magnetometers. The proposed drone positioning algorithm takes advantage of the UWB positioning accuracy and of the higher sampling rate of the IMUs and provides accurate estimates of the position of the drone, even when the drone suffers from high accelerations. This positioning algorithm is executed in the single board computer (SBC) of the drone and works in two steps. In the first step, for each tag, the proposed drone positioning algorithm fuses the information of the IMU and magnetometer with UWB data to estimate its position. In the second step, the positioning estimates of each tag are combined to provide a more accurate estimate of the position of the centre of the drone. Unlike other solutions in the state of art, our proposal neither needs a complex infrastructure deployment, nor does it depend on lighting conditions or availability of GNSS. Additionally, our proposed system presents high accuracy even with sudden changes in drone velocity, as it achieves a higher positioning rate than traditional UWB-based positioning systems. Finally, the proposed combination of tags’ positions further improves the accuracy of our system. Higher robustness is gained because the possible errors of a tag are compensated with the other.

The rest of the article is organised as follows: Section 2 describes how a LAS works when only UWB data are used, Section 3 describes the proposed LAS and the main contributions to the state of art, Section 4 explains the performed experiments and analysis and the obtained results are presented in Section 5. Finally, conclusions and future research lines are given in Section 6.

## 2. State of the Art of UWB-Based Systems

When UWB technology is used as RTLS, two main elements are necessary: anchors and tags. Anchors are fixed sensors at known locations, while tags are the moving sensors to be located. Each tag communicates with the anchors in order to calculate the distance to all of them. With the measured distances and the known locations of anchors, the positions of the tags can be calculated.

For the real-time location of a UAV, a single tag is usually placed on the vehicle and an anchor infrastructure is deployed around the flying space. Ideally, the anchors should have a separation of tens of meters so that the calculation is optimal. This type of infrastructure is typical in the literature, as proposed for example by [37,38,39]. Nevertheless, optimal anchor infrastructures cannot always be deployed, so [42] suggests a deployment where four anchors are placed on a 2 × 2 m square on the floor.

**Figure 1:**
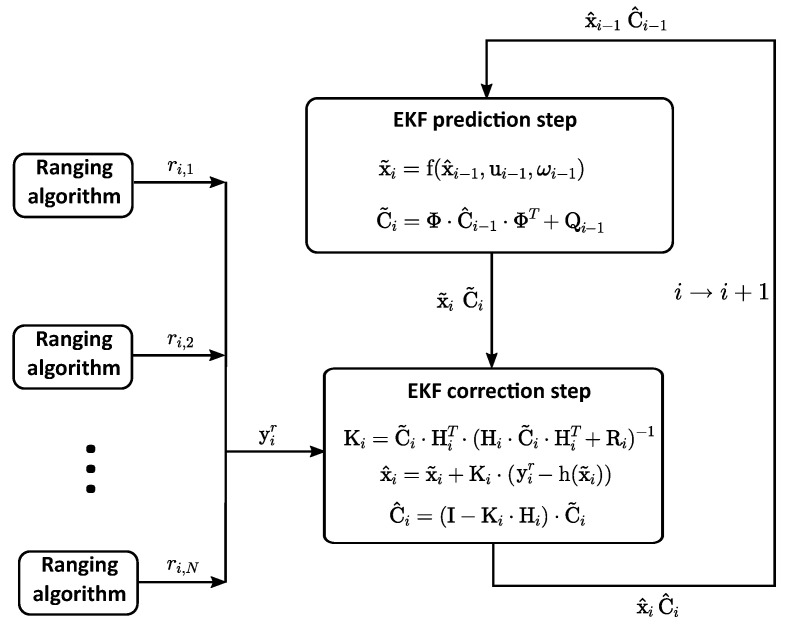
Flow chart of an extended Kalman filter (EKF) with ultra-wideband (UWB) sensors for localisation.

With an anchor infrastructure and a tag on the drone, the estimated distances can be used in different algorithms to calculate the position of the vehicle. One of the most typical methods to calculate the position of a tag from ranging measurements to known anchors is the Extended Kalman Filter (EKF). An algorithm based on the EKF is used by [41,55], which is shown in Figure 1.

This algorithm needs a motion model f and an observation model h to be defined

(1) $$\begin{array}{cc} {\tilde{x}}_{i} & =f({\hat{x}}_{i-1},u_{i-1},\omega_{i-1}) \end{array}$$

(2) $$\begin{array}{cc} {\tilde{y}}_{i} & =h({\tilde{x}}_{i},\nu_{i}), \end{array}$$

where $x_{i}$ is the state vector related to the ith estimation. It contains the position of the tag to be estimated and its first and second derivatives

(3) $$x_{i}={\left[\begin{array}{ccccccccc} x_{i} & {\dot{x}}_{i} & {\ddot{x}}_{i} & y_{i} & {\dot{y}}_{i} & {\ddot{y}}_{i} & z_{i} & {\dot{z}}_{i} & {\ddot{z}}_{i} \end{array}\right]}^{T},$$

$u_{i}$ is the optional input vector set to zero and f is the function representing the motion model of the system. It relates the previous state $x_{i-1}$ with the current state $x_{i}$. The observation vector is represented by $y_{i}$, which contains the measured distances between the tag and each anchor. These distances can be calculated with the state estimate $x_{i}$ and the function of the observation model h. Note that $\tilde{x}$ and $\hat{x}$ notation in (1) represents the *a priori* and *a posteriori* state estimate, respectively. The process is characterised with the stochastic random variables $\omega_{i}$ and $\nu_{i}$ that represent the process and observation noise, respectively. They are assumed to be independent, white and normal probably distributed with covariance matrices $Q_{i}$ and $R_{i}$, respectively.

The above mentioned *a priori* estimate of the state is calculated with the linearised version of the motion model f

(4) $$\begin{array}{cc} {\tilde{x}}_{i} & =\Phi \cdot {\hat{x}}_{i-1} \end{array}$$

(5) $$\begin{array}{cc} \Phi & =I_{3\times 3}⊗B \end{array}$$

(6) $$\begin{array}{cc} B & =\left[\begin{array}{ccc} 1 & \Delta & \frac{\Delta^{2}}{2}\\ 0 & 1 & \Delta \\ 0 & 0 & 1 \end{array}\right] \end{array}$$

(7) $$\begin{array}{cc} {\tilde{C}}_{i} & =\Phi \cdot {\hat{C}}_{i-1}\cdot \Phi^{T}+Q_{i-1}, \end{array}$$

where ⊗ represents the Kronecker product of the matrices, Δ the time difference between two consecutive time steps and C the error covariance matrix of the state estimate.

Using the predicted estimate of the state vector, the predicted observation vector ${\tilde{y}}_{i}$ can be calculated by means of the observation model h. For each anchor *l*, the distance between the predicted position ${\left({\tilde{x}}_{i},{\tilde{y}}_{i},{\tilde{z}}_{i}\right)}^{T}$ and the fixed sensor position ${\left(X_{l},Y_{l},Z_{l}\right)}^{T}$ is calculated as

(8) $${\tilde{y}}_{i,l}=\sqrt{{\left({\tilde{x}}_{i}-X_{l}\right)}^{2}+{\left({\tilde{y}}_{i}-Y_{l}\right)}^{2}+{\left({\tilde{z}}_{i}-Z_{l}\right)}^{2}}.$$

Finally, the predicted state ${\tilde{x}}_{i}$ is corrected to obtain ${\hat{x}}_{i}$ by comparing the predicted observation vector ${\tilde{y}}_{i}$ with the measured ranging values $y_{i}^{r}$

(9) $$\begin{array}{cc} {\hat{x}}_{i} & ={\tilde{x}}_{i}+K_{i}\cdot \left(y_{i}^{r}-{\tilde{y}}_{i}\right) \end{array}$$

(10) $$\begin{array}{cc} K_{i} & ={\tilde{C}}_{i}\cdot H_{i}^{T}\cdot {\left(H_{i}\cdot {\tilde{C}}_{i}\cdot H_{i}^{T}+R_{i}\right)}^{-1} \end{array}$$

(11) $$\begin{array}{cc} H_{i} & =\frac{\partial h}{\partial x}\left({\tilde{x}}_{i}\right) \end{array}$$

(12) $$\begin{array}{cc} {\hat{C}}_{i} & =\left(I-K_{i}\cdot H_{i}\right)\cdot {\tilde{C}}_{i}, \end{array}$$

where $H_{i}$ is the Jacobian matrix of the observation model h.

Despite the high accuracy of the UWB technology and the suitability of the EKF for a correct performance of this type of locating systems, they still present some drawbacks for the UAV localisation. The data rate of UWB systems is limited and may be incapable of detecting sudden changes in the drone path due to sudden wind changes. In order to deal with these types of conditions, it is better to add the data from an IMU that could give an accurate estimate of the vehicle’s acceleration, track all the trajectory changes and improve the data rate of the position estimates.

## 3. Proposed LAS

In this section the proposed novel LAS and its main differences compared to the typical UWB-based systems described in Section 2 are explained. Note that the proposed LAS is designed for a drone that inspects critical infrastructures such as off-shore wind turbines or a tank of a petrochemical plant. After the inspection mission, the drone needs to land on a small platform to charge its batteries. This platform is on a boat or in a confined space, so there is not enough space or time to deploy typical UWB infrastructure. In our proposal, a small anchor infrastructure with easy deployment is used, which also allows us to make the UWB anchors part of the landing platform and use the same power supply for the anchors and the battery charger. Thus, the resulting LAS needs no complex additional infrastructure. Moreover, our LAS is not affected by changing lighting conditions because of the day or night time, rain or fog that traditionally affect computer-vision systems. As our system is based on UWB technology, during the landing, our LAS will present lower positioning errors than GNSS, which can be around 2 m in the latter case [29].

**Figure 2:**
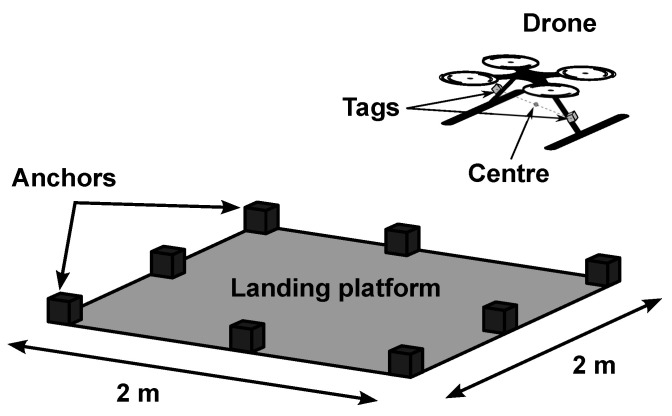
Proposed system architecture. Eight anchors are placed around the landing platform in order to locate the tags on the drone in real-time.

In this work, data from UWB, IMU and magnetometer are proposed to be combined to estimate the position of the drone. Figure 2 depicts the system architecture.

Similar to [42], eight anchors are placed around the landing platform of 2 × 2 m and two tags are installed on both sides of the drone. Taking advantage of the availability of these sensors, a new positioning algorithm is proposed. This algorithm first fuses the UWB, IMU and magnetometer data from each tag to obtain two independent position estimates and then combines them to calculate the position of the centre of the drone. From the resulting data, only the horizontal coordinates of the drone are used since the vehicle is capable of accurately estimating its altitude with other sensors; i.e., an altimeter.

**Figure 3:**
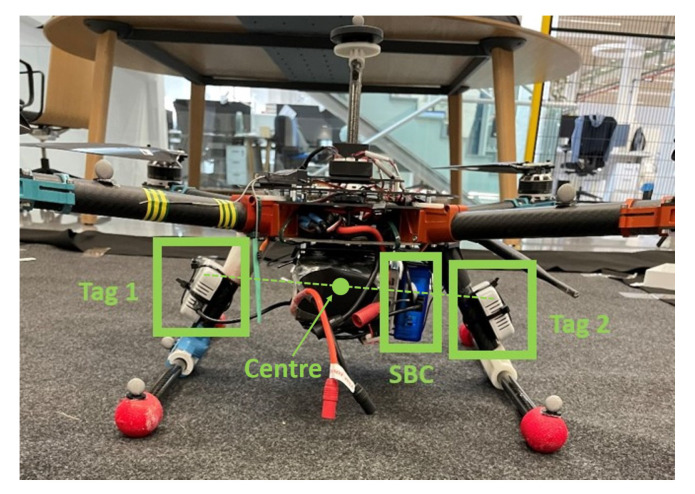
Tags on the drone.

Figure 3 shows the placement of the tags on the drone.

They are installed on both sides of the drone with a separation of 0.36 m. In this work, the LAS will provide the position of the point that is in the middle of the line formed by the two tags. We will denote the centre of the drone to this point. In the same picture, the SBC of the drone can be seen, which receives the data from both tags and runs the necessary positioning algorithm.

The tags employ the DW1000 chip of Decawave as UWB transceiver. This transceiver follows the IEEE 802.15.4a standard and is configured with the parameters presented in Table 1.

Apart from the UWB transceiver, the tags also contain the LSM6DSOTR IMU [56] and the LIS2MDLTR magnetometer [57]. Both sensors are developed by STMicroelectronics and they can be fused in the MotionFX library of STMicroelectronics [58] in order to subtract the measurement of gravity from the acceleration data and obtain the orientation of the tag. The chosen configuration parameters of MotionFX are shown in Table 2.

**Figure 4:**
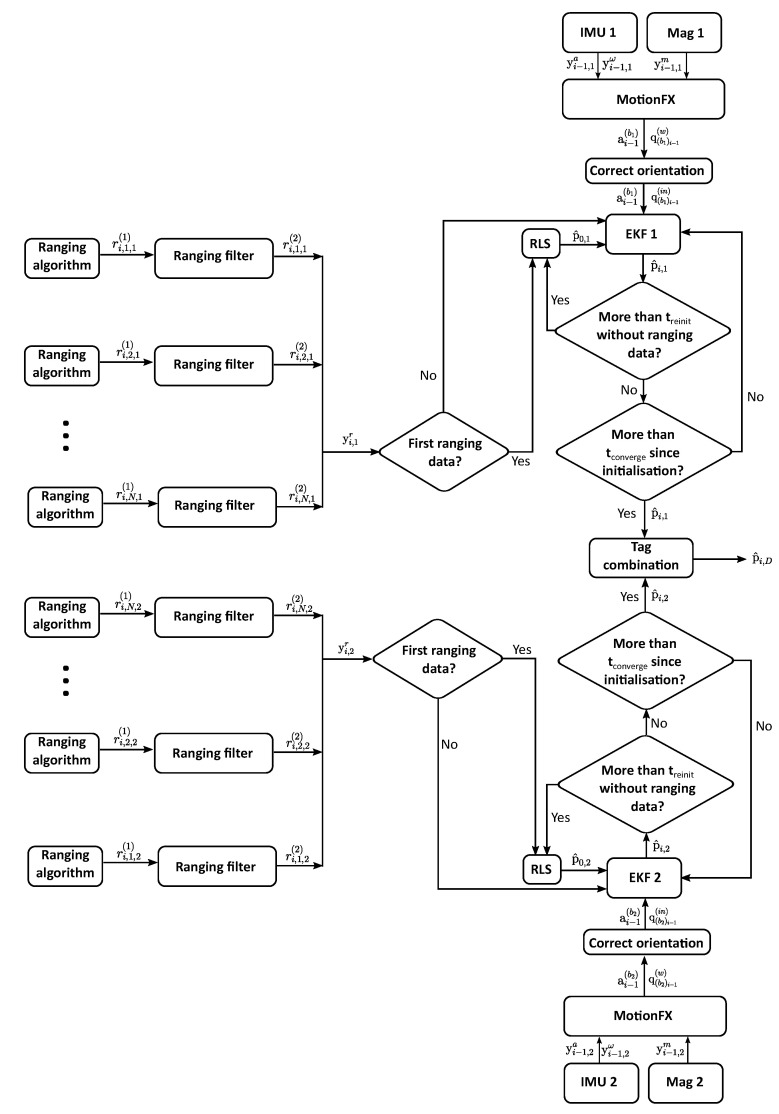
Flow chart of the positioning algorithm.

Figure 4 shows the flow chart of the proposed algorithm. As described in Section 2, the tags communicate with the anchors in order to calculate the distances between the sensors, $r_{i,l,j}^{\left(1\right)}$, using the two way ranging (TWR) method. The subscripts *i*, *l* and *j* of the ranging estimates refer to the time step, the identifier of the anchor and the identifier of the tag, respectively. The obtained data are sent to the SBC of the drone (see Figure 3) which runs the necessary algorithms for a correct position estimation.

Unlike the state of art, our LAS filters the ranging estimates with a parameter $r_{max}$, which represents the maximum allowed ranging estimate. Since the objective of the proposed LAS is to help the drone during the autonomous landing and not the rest of the flight, any estimate ranging above $r_{max}$ is discarded.

**Figure 5:**
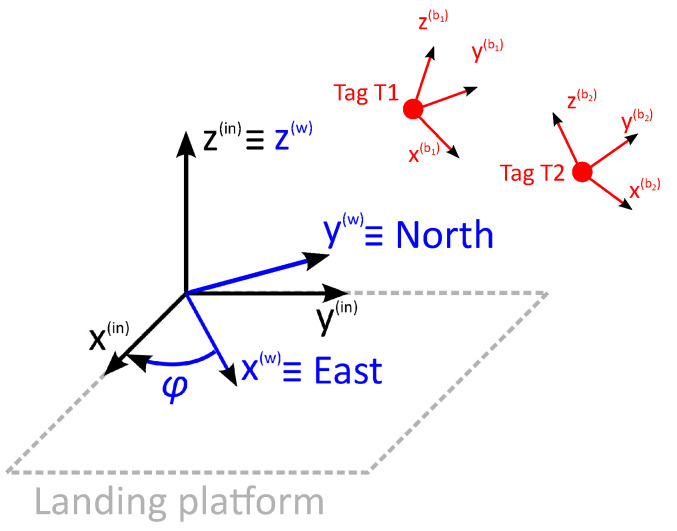
Body, world and inertial frames.

Moreover, our proposed LAS adds the data of two IMUs and magnetometers to the algorithm, one for each tag. At time $t_{i-1}$ and tag *j*, the measured specific force $y_{i-1,j}^{a}$, angular velocity $y_{i-1,j}^{\omega }$ and magnetic field $y_{i-1,j}^{m}$ are used by the MotionFX library to calculate the acceleration $a_{i-1}^{\left(b_{j}\right)}$ and quaternion $q_{{\left(b_{j}\right)}_{i-1}}^{\left(w\right)}$. The terms $\left(b_{j}\right)$ and (w) refer to the body frame of tag *j* and world frame, respectively. The used frames are shown in Figure 5.

Each tag contains an independent body frame, $\left(b_{1}\right)$ and $\left(b_{2}\right)$, which are fixed to the sensors. All measurements of the IMUs and magnetometers and the resulting acceleration $a_{i-1}^{\left(b_{j}\right)}$ are referred to their body frames. The quaternion $q_{{\left(b_{j}\right)}_{i-1}}^{\left(w\right)}$ transforms any vector referred to the body frame $\left(b_{j}\right)$ to the world frame (w), of which the *x*, *y* and *z* axes look at the east, north and up, respectively. However, the inertial frame (in), which is defined by the landing platform, does not have to be aligned with the world frame, so another quaternion $q_{\left(w\right)}^{\left(in\right)}$ must be defined to transform any vector referred to the world frame (w) to the inertial frame (in). If the landing platform is on the horizontal plane, $q_{\left(w\right)}^{\left(in\right)}$ is defined as a quaternion that rotates any vector by an angle ϕ around the *z* axis. Both quaternions can be combined to calculate the quaternion $q_{{\left(b_{j}\right)}_{i-1}}^{\left(in\right)}$ that transforms any vector from the body frame $\left(b_{j}\right)$ to the inertial frame (in)

(13) $$q_{{\left(b_{j}\right)}_{i-1}}^{\left(in\right)}=q_{\left(w\right)}^{\left(in\right)}⊙q_{{\left(b_{j}\right)}_{i-1}}^{\left(w\right)},$$

being ⊙ the quaternion multiplication operator.

The calculated ranging data, acceleration and orientation of the tags are fused in two parallel EKF algorithms. This way, two position estimates ${\hat{p}}_{i,1}$ and ${\hat{p}}_{i,2}$ are calculated and finally combined to obtain the position of the centre of the drone ${\hat{p}}_{i,D}$. If for some reason one of the tags does not see the anchors for a time $t_{reinit}$, that tag stops giving position estimates. In this case, the position of the centre of the drone can still be calculated with the position estimate of the other tag, its orientation and the relative position of the centre of the drone with respect to the remaining tag. Once the UWB signal is available again in the tag, its EKF is reinitialised. This means that all the parameters of the EKF are set to their initial value. As the EKF algorithm needs some time to converge to the real solution, during a time period of $t_{converge}$, the position estimates of the newly found tag are not used in the combination algorithm. The EKF algorithm is further explained in Section 3.1 and the combination algorithm in Section 3.2.

### 3.1. EKF with Fusion of Sensors

**Figure 6:**
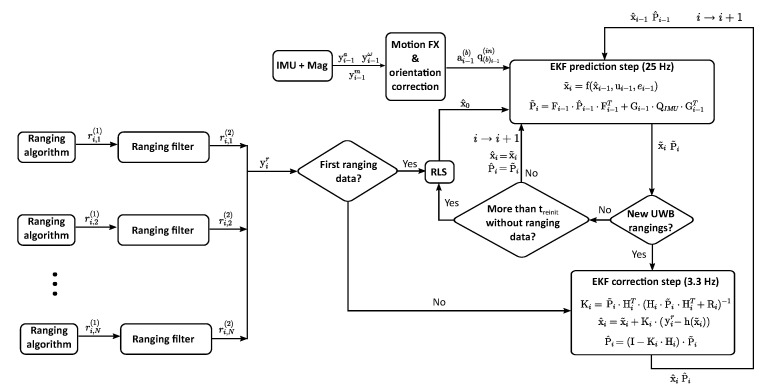
Flow chart of the EKF that fuses the UWB data with an inertial measurement unit (IMU) and magnetometer.

The first part of the proposed positioning algorithm consists of an EKF that takes advantage of the availability of the data of the IMU and magnetometer. The flow chart that summarises this part is shown in Figure 6.

After a reinitialisation, the first position of the tag is estimated by means of a recursive least squares (RLS) algorithm [59] using the first received UWB data. After this first position estimate, every time a new acceleration estimate is received, the prediction step is performed. Since the IMU and UWB rates are different, while no new UWB measurements are received, the EKF algorithm keeps working with the predicted state estimate. When new UWB data are received, the correction step is performed. The advantage of this method is that the resulting positioning rate of the proposed LAS is of 25 Hz, much faster than the UWB ranging rate. However, if no UWB measurements are obtained for a long period of time, the position estimate can drift and get lost. For this reason, if not enough UWB ranging estimates are obtained during an adjustable time interval of $t_{reinit}$, the proposed LAS stops giving position estimates. Once the UWB signal is recovered, the algorithm is reinitialised.

This algorithm is run twice in parallel, once for each tag. For simplicity, the letter *j* that is used to refer to the tag is going to be skipped in this subsection.

Unlike the previous algorithm of Section 2, the state vector only contains position and velocity data

(14) $$x_{i}={\left[\begin{array}{cc} p_{i}^{T} & v_{i}^{T} \end{array}\right]}^{T}={\left[\begin{array}{cccccc} x_{i} & y_{i} & z_{i} & {\dot{x}}_{i} & {\dot{y}}_{i} & {\dot{z}}_{i} \end{array}\right]}^{T},$$

with $p_{i}$ being the position of the tag at time step *i* and $v_{i}$ its velocity. The acceleration data are introduced in the motion model as one of the input parameters. The inputs are the acceleration referred to the body frame $a^{\left(b\right)}$ and a unit quaternion $q_{\left(b\right)}^{\left(in\right)}$ that rotates any vector from the body frame (b) to the inertial frame (in).

The motion model f that transforms the previous state ${\hat{x}}_{i-1}={\left[\begin{array}{cc} {\hat{p}}_{i-1}^{T} & {\hat{v}}_{i-1}^{T} \end{array}\right]}^{T}$ to the current predicted state ${\tilde{x}}_{i}={\left[\begin{array}{cc} {\tilde{p}}_{i}^{T} & {\tilde{v}}_{i}^{T} \end{array}\right]}^{T}$ is defined as

(15) $$\begin{array}{cc} {\tilde{x}}_{i} & =f\left({\hat{x}}_{i-1},u_{i-1},e_{i-1}\right) \end{array}$$

(16) $$\begin{array}{cc} u_{i-1} & ={\left[\begin{array}{cc} a_{i-1}^{\left(b\right)} & q_{{\left(b\right)}_{i-1}}^{\left(in\right)} \end{array}\right]}^{T} \end{array}$$

(17) $$\begin{array}{cc} e_{i-1} & ={\left[\begin{array}{cc} e_{i-1}^{\left(a\right)} & e_{i-1}^{\left(\phi \right)} \end{array}\right]}^{T} \end{array}$$

(18) $$\begin{array}{cc} \;{\tilde{p}}_{i} & ={\hat{p}}_{i-1}+\Delta \cdot {\hat{v}}_{i-1}+\frac{\Delta^{2}}{2}\cdot R_{{\left(b\right)}_{i-1}}^{\left(in\right)}\cdot \left(a_{i-1}^{\left(b\right)}-e_{i-1}^{\left(a\right)}\right) \end{array}$$

(19) $$\begin{array}{cc} {\tilde{v}}_{i} & ={\hat{v}}_{i-1}+\Delta \cdot R_{{\left(b\right)}_{i-1}}^{\left(in\right)}\cdot \left(a_{i-1}^{\left(b\right)}-e_{i-1}^{\left(a\right)}\right) \end{array}$$

(20) $$\begin{array}{cc} R_{{\left(b\right)}_{i-1}}^{\left(in\right)} & =q2R\left(q_{{\left(b\right)}_{i-1}}^{\left(in\right)}⊙f_{q}\left(e_{i-1}^{\left(\phi \right)}\right)\right), \end{array}$$

where Δ represents the time between two consecutive steps and $R_{\left(b\right)}^{\left(in\right)}$ the rotation matrix obtained from the unit quaternion $q_{\left(b\right)}^{\left(in\right)}$ as explained in [60] with the here defined function q2R. For any unit quaternion $q={\left[\begin{array}{cccc} q_{w} & q_{x} & q_{y} & q_{z} \end{array}\right]}^{T}$, its corresponding rotation matrix $R_{q}$ is calculated as

(21) $$R_{q}=q2R\left(q\right)=\left[\begin{array}{ccc} 2q_{w}^{2}+2q_{x}^{2}-1 & 2q_{x}q_{y}-2q_{w}q_{z} & 2q_{x}q_{z}+2q_{w}q_{y}\\ 2q_{x}q_{y}+2q_{w}q_{z} & 2q_{w}^{2}+2q_{y}^{2}-1 & 2q_{y}q_{z}-2q_{w}q_{x}\\ 2q_{x}q_{z}-2q_{w}q_{y} & 2q_{y}q_{z}+2q_{w}q_{x} & 2q_{w}^{2}+2q_{z}^{2}-1 \end{array}\right].$$

The noise parameters of the motion model f are represented with $e^{\left(a\right)}$ for the acceleration data and $e^{\left(\phi \right)}$ for the orientation data. The latter is represented as an orientation deviation in the body coordinate frame and it is converted to a unit quaternion $q^{\left(\phi \right)}$ with the function $f_{q}$

(22) $$q^{\left(\phi \right)}=f_{q}\left(e^{\left(\phi \right)}\right)=\left[\begin{array}{c} \cos(\frac{\left|\right|e^{\left(\phi \right)}{\left|\right|}_{2}}{2})\\ \frac{e^{\left(\phi \right)}}{\left|\right|e^{\left(\phi \right)}{\left|\right|}_{2}}\sin\left(\frac{\left|\right|e^{\left(\phi \right)}{\left|\right|}_{2}}{2}\right) \end{array}\right],$$

being $\left|\right|e^{\left(\phi \right)}{\left|\right|}_{2}$ the euclidean norm of vector $e^{\left(\phi \right)}$.

Both noise parameters are determined empirically and have zero mean and covariance $Q_{IMU}$, which is necessary for the prediction step of the EKF to make an *a priori* estimation of the state error covariance matrix ${\tilde{P}}_{i}$

(23) $$\begin{array}{cc} {\tilde{P}}_{i} & =F_{i-1}\cdot {\hat{P}}_{i-1}\cdot F_{i-1}^{T}+G_{i-1}\cdot Q_{IMU}\cdot G_{i-1}^{T} \end{array}$$

(24) $$\begin{array}{cc} F_{i-1} & =\frac{\partial f}{\partial x}\left({\hat{x}}_{i-1}\right) \end{array}$$

(25) $$\begin{array}{cc} G_{i-1} & =\frac{\partial f}{\partial e}\left(e_{i-1}\right). \end{array}$$

In the above equation the Jacobian matrices $F_{i-1}$ and $G_{i-1}$ of the motion model f have been calculated with respect to the state vector x and noise vector e. The calculation process of useful derivatives for quaternions and rotation matrices is explained in [61].

After the prediction step, the *a priori* estimate must be corrected with the UWB ranging data as

(26) $${\hat{x}}_{i}={\tilde{x}}_{i}+K_{i}\cdot \left(y_{i}^{r}-{\tilde{y}}_{i}\right),$$

where $y_{i}^{r}$ is the vector of measured ranging values, ${\tilde{y}}_{i}$ is the predicted observation vector calculated with (8) and $K_{i}$ represents the Kalman gain matrix. The Kalman gain matrix is calculated as

(27) $$K_{i}={\tilde{P}}_{i}\cdot H_{i}^{T}\cdot {\left(H_{i}\cdot {\tilde{P}}_{i}\cdot H_{i}^{T}+R_{i}\right)}^{-1},$$

where $H_{i}$ is the Jacobian matrix of the observation model and $R_{i}$ the measurement covariance matrix. The Jacobian matrix of the observation model is calculated with (11). Finally, the predicted state error covariance matrix ${\tilde{P}}_{i}$ must be corrected with

(28) $${\hat{P}}_{i}=\left(I-K_{i}\cdot H_{i}\right)\cdot {\tilde{P}}_{i}.$$

### 3.2. Combination of Tags

In the last part of the proposed positioning algorithm, the two independent position estimates ${\hat{p}}_{i,1}$ and ${\hat{p}}_{i,2}$ are combined to calculate the position of the centre of the drone ${\hat{p}}_{i,D}$. If the estimates of both tags are available at time step *i*, then the average position is calculated. If at some certain moment, only one of the tags gives a positioning estimate, then the position of the centre of the drone can be calculated with the known orientation $q_{{\left(b_{j}\right)}_{i}}^{\left(in\right)}$ and the coordinates of the centre of the drone $d_{j}$ with respect to the body frame of the remaining tag $\left(b_{j}\right)$. The algorithm is summarised in (29)

(29) $${\hat{p}}_{i,D}=\left\{\begin{array}{cc} \frac{{\hat{p}}_{i,1}+{\hat{p}}_{i,2}}{2}, & \mathrm{if}\;\exists {\hat{p}}_{i,1},\exists {\hat{p}}_{i,2}\\ {\hat{p}}_{i,1}+q2R\left(q_{{\left(b_{1}\right)}_{i}}^{\left(in\right)}\right)\cdot d_{1}, & \mathrm{if}\;\exists {\hat{p}}_{i,1},∄{\hat{p}}_{i,2}\\ {\hat{p}}_{i,2}+q2R\left(q_{{\left(b_{2}\right)}_{i}}^{\left(in\right)}\right)\cdot d_{2}, & \mathrm{if}\;∄{\hat{p}}_{i,1},\exists {\hat{p}}_{i,2}. \end{array}\right.$$

## 4. Methodology

For the correct assessment of the proposed LAS, some experiments were performed by flying the drone in a controlled indoor environment close to the landing area. Additionally, more experiments were conducted in a real outdoor environment. In both cases, the parameters $r_{max}$, $t_{reinit}$ and $t_{converge}$ described in Section 3 were set to 20 m, 2 s and 3 s, respectively. In the next subsections, the experimental set-ups as well as the employed evaluation methods are described.

### 4.1. Indoor Experiments

**Figure 7:**
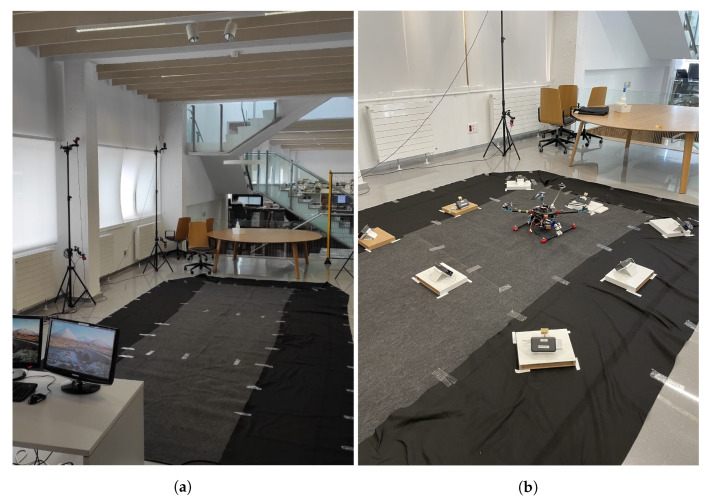
(**a**) Location of the tests and (**b**) set-up for the measurements.

All the indoor tests were run in the Industry 4.0 Laboratory of Ceit-BRTA which contains a motion capture system of Optitrack, which allowed us to track the drone with millimetre level accuracy. Due to the high accuracy of the motion capture system, its measurements were used as ground truth. A picture of the testing zone can be seen in Figure 7a. The developed LAS was deployed inside the observation area of the Optitrack system, as shown in Figure 7b and the positions of each anchor are given in Table 3.

For safety reasons, some fences were placed around the measurement zone.

**Figure 8:**
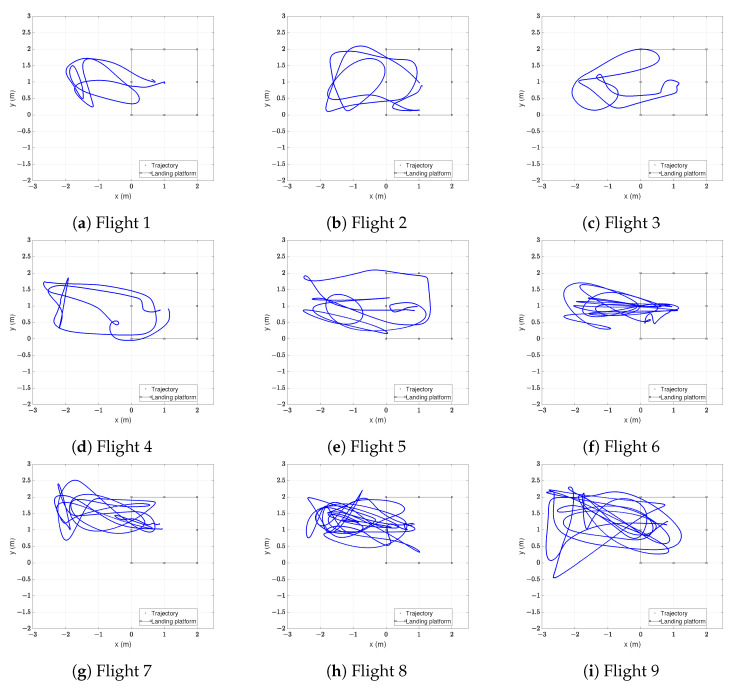
Horizontal trajectory of each flight. Blue line represents the movements of the drone and the black-edged square the landing platform.

Once the set-up was prepared, 9 different flights were conducted inside the tracking area. All of them consisted of a take-off, movements close to the landing platform and a landing. The paths followed by the centre of the drone during the flights can be seen in Figure 8.

The flights can be separated into two groups: those with a mean horizontal acceleration under 1 $m/s^{2}$ and those with a mean horizontal acceleration over 1 $m/s^{2}$, as shown in Table 4.

The measured accelerations correspond to the centre of the drone, so the acceleration on each tag may be slightly different. By dividing the flights in two groups, the effect of acceleration on the positioning accuracy can be evaluated.

### 4.2. Outdoor Experiments

**Figure 9:**
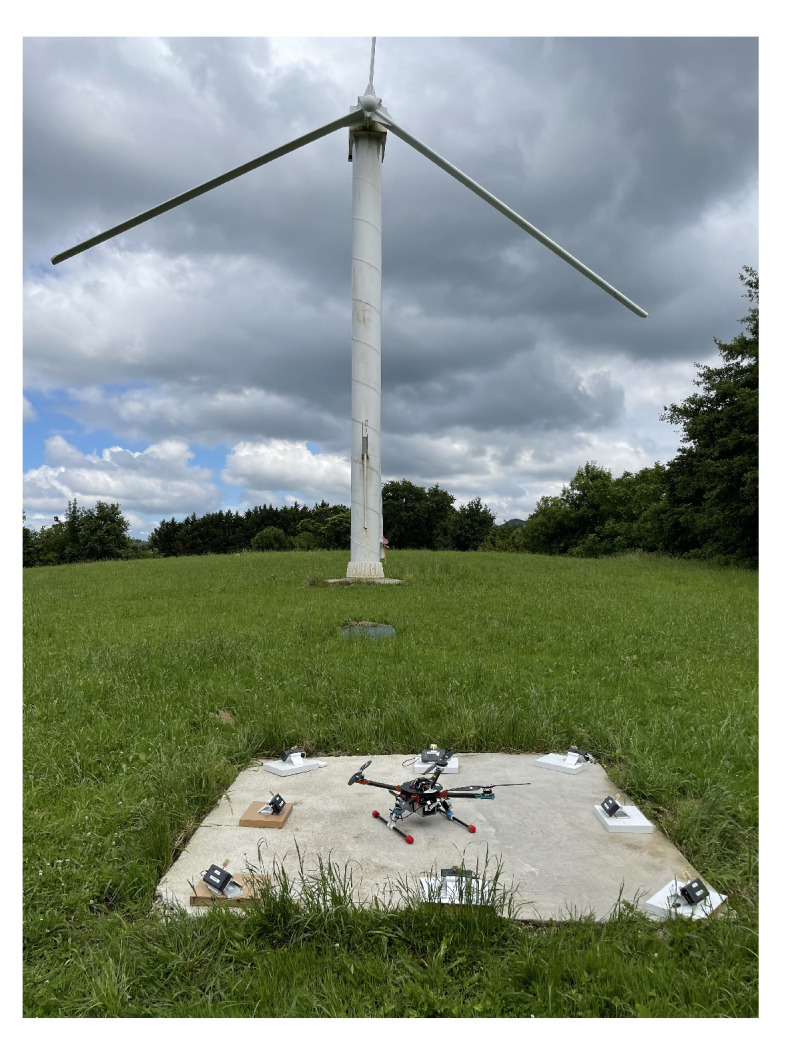
Set-up in the outdoor environment.

The drone was also flown in a real outdoor environment with the proposed LAS. A picture of the test zone is shown in Figure 9 with the prepared set-up.

The anchors were placed in the same positions as described in Table 3. These experiments were useful to test the LAS at longer distances than in the indoor environment. It is especially interesting to test the ability of the LAS to find the drone once it reaches the visible range of the system, when the landing is about to occur.

**Figure 10:**
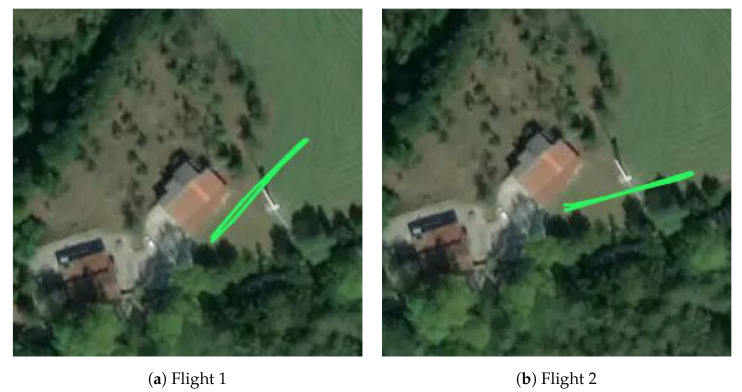
Horizontal trajectory of each flight in the outdoor environment.

The chosen place contains a concrete platform of 2 × 2 m to land the drone and deploy the LAS. There is also a wind turbine, which simulates the infrastructure that the drone should inspect. Two different flights were performed, both of which consisted of a take-off, a linear movement to the wind turbine reaching a height of 14 m, return and landing, as shown in Figure 10.

Because of the unavailability of a highly accurate outdoor locating system such as Optitrack, the performance of the system in this environment was evaluated qualitatively by comparing it to the GNSS position estimates.

### 4.3. Calculation of Errors

In order to evaluate the performance of the proposed system, the positioning error in the horizontal plane XY was calculated as

(30) $$\epsilon_{i}=\sqrt{{\left({\hat{x}}_{i}-x_{i}\right)}^{2}+{\left({\hat{y}}_{i}-y_{i}\right)}^{2}},$$

where $\epsilon_{i}$ represents the error of the position estimate *i*, $x_{i}$ and $y_{i}$ the real 2D position coordinates and ${\hat{x}}_{i}$ and ${\hat{y}}_{i}$ the estimated 2D position.

Once all the positioning errors were calculated, the system was evaluated with the mean error μ, standard deviation σ and root mean square error RMSE [62]. Additionally, the error below which 80% of samples are, the probability of obtaining an error under 1 m and the maximum error $\epsilon_{max}$ were calculated.

## 5. Results

In this section the obtained experimental results are presented and discussed. As explained in the previous section, two types of measurements were performed. The first group was in an indoor controlled environment with the objective of evaluating the feasibility of the proposed LAS. The performance of the system was evaluated with only UWB data and later with the fusion of the inertial data, so that the advantages of data fusion could be seen. The second group of experiments was performed in a realistic environment, and the performance of the proposed LAS was qualitatively evaluated.

### 5.1. Indoor Results

#### 5.1.1. Accuracy with Four UWB Anchors

For a correct comparison with the systems of the state of art, the performance of our LAS was evaluated using only UWB data. First, a similar set-up to that proposed by [42] was considered; i.e., only the ranging estimates of the four anchors on the corners were used to position the tags.

In Table 5, the accuracy data are given for both tags.

These tables show, for each flight, the mean positioning error, its standard deviation, the root mean square error, the error below which 80% of samples are, the percentage of errors below 1 m and the measured maximum error.

**Figure 11:**
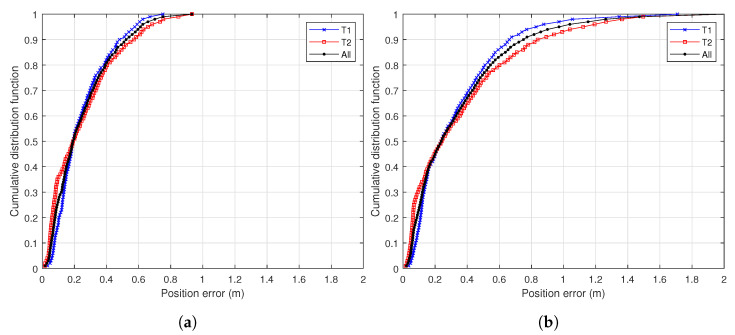
Cumulative distribution function plots of (**a**) Flights 1–4 and (**b**) Flights 5–9.

The obtained results confirm that a small anchor infrastructure of 2 × 2 m can accurately locate a drone when it flies close to the landing platform. Considering all flights, the RMSE value was 0.377 m for Tag T1 and 0.442 m for Tag T2. However, there was a considerable difference between those flights with low horizontal acceleration (Flights 1 to 4) and those with high acceleration (Flights 5 to 9). This is confirmed with the cumulative distribution function plots shown in Figure 11a for Flights 1 to 4 and Figure 11b for Flights 5 to 9.

When the acceleration values were low, as can be seen in Figure 11a, the obtained results were similar to those of [42]. In this case, the authors of [42] measured a mean horizontal acceleration of 0.67 $m/s^{2}$ and a maximum of 2.35 $m/s^{2}$. However, our results demonstrate that when the drone suffered a higher acceleration (Flights 5 to 9) the accuracy of the UWB-based LAS was reduced, so a traditional system using only UWB data could have problems under adverse conditions.

#### 5.1.2. Accuracy with Eight UWB Anchors

For a better performance of the LAS, our proposal adds some redundancy by using the estimates of eight anchors instead of four. The benefit of having anchor redundancy is that it is possible to calculate new positions even if an anchor fails to see the tags. If only four anchors are used, the lack of a single anchor-tag distance measurement is enough to skip a new position sample. With eight anchors, however, new positions can be calculated even with the lack of four sensors’ measurements. We tested the effect of this redundancy on the positioning accuracy and Table 6 shows the obtained data for the LAS using only UWB data with eight anchors.

The added redundancy reduced the mean error, RMSE and especially the maximum error. However, just adding more anchors could not solve the problems in the flights of higher accelerations. These flights need a high sampling rate sensor such as an IMU, as we propose in our LAS.

#### 5.1.3. Accuracy with Fusion of Data

With the data of eight UWB anchors, an IMU and a magnetometer, our proposed LAS uses the EKF algorithm presented in Section 3.1 to fuse all this information. In Table 7 the obtained results of this algorithm are shown when it is used to estimate the position of the two tags of the drone.

Compared to the obtained results with only UWB data of eight anchors, the data fusion improved the accuracy of the system, especially in the second group of flights, where the mean horizontal acceleration was over 1 $m/s^{2}$. For example, significant changes can be noticed in Flight 5 with the data fusion algorithm: the position of Tag T1 had an RMSE of 0.194 m and Tag T2 had an RMSE of 0.249 m. Without the proposed fusion algorithm, these values were 0.401 m and 0.503 m, respectively, so our proposed positioning algorithm halved the RMSE values in this case. Furthermore, the maximum error in this flight also had a reduction of around 50% in both tags. In general, our proposal significantly reduced the values of the mean error, standard deviation and maximum error in all flights with high accelerations.

Moreover, the fusion of data was also beneficial for those flights with low accelerations as almost all error metrics of every flight improved. Considering all data, with our proposed fusion algorithm, great accuracy can be obtained to locate a drone close to its landing platform.

#### 5.1.4. Accuracy with a Combination of Tags

Finally, our proposed LAS combines both tags of the drone for a more accurate path. After fusing the UWB data from each tag with their IMUs, both position estimates are fused to calculate the position of the centre of the drone. In Table 8 the accuracy of the proposed system is shown.

Compared to the individual results of Table 7, the accuracy was further improved. The mean error and the RMSE were reduced in almost all cases. Moreover, there was a general reduction of the standard deviation of the error, which means a reduction of outliers. When only a tag was used to estimate positions, it could sometimes be with an unfavourable orientation with respect to the anchors. In these cases, the position estimates would suffer from high errors. With two tags, it is less likely to have a bad estimate at the same time with both of them. Therefore, the biggest errors were compensated with the help of the other tag. Thus, significant improvements can also be seen in the percentage of samples with an error under 1 m and in the maximum error.

#### 5.1.5. Summary of Results

As a summary of the improved results, Table 9 shows the key metrics obtained with a set-up similar to [42] and with our proposed LAS.

The first row is the result of considering all the measured errors of both tags and all flights when positioning with only UWB data of four anchors. We can observe that the proposed LAS has improved all performance metrics. The reduction in RMSE value is remarkable, as it reduced from 0.410 m to 0.208 m; i.e., our novel LAS can reduce the obtained errors by 50% compared to a typical system. Thanks to a higher accuracy and a more frequent data rate, the task of autonomous landing becomes much safer with our proposal.

### 5.2. Results in a Real Environment

In addition to the indoor measurements, the proposed LAS was also tested in an outdoor realistic environment. In this way, it can be assessed how the LAS finds the drone after the inspection mission and how it tracks the vehicle until the landing manoeuvre.

**Figure 12:**
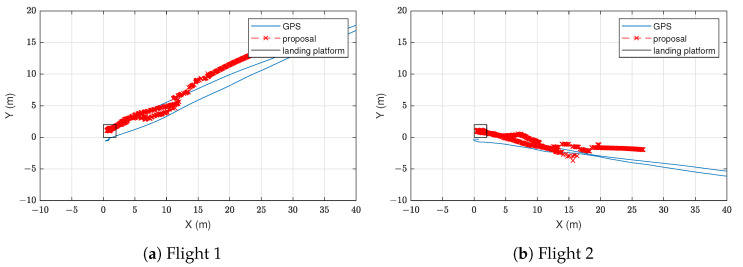
Estimated trajectory in the outdoor environment by the combination of tags. Estimates of the proposed landing assistance system (LAS) are given in red colour. Blue line represents the trajectory given by the global navigation satellite system (GNSS) of the drone.

Figure 12 represents the estimated trajectories by the proposed LAS compared to the GNSS. The position estimates of our proposal are shown as red points, while the GNSS trajectories are represented as blue lines. However, these GNSS data could not be used as ground truth, since their errors were similar to or greater than those of the UWB system. As an example, note that in both flights the GNSS incorrectly estimated that the drone landed out of the platform, while the proposed system is able to correctly estimate the landing place.

Due to dilution of precision, the accuracy of a UWB positioning system such as the one proposed in [42] is degraded at large distances. However, thanks to the information of the IMUs and magnetometers, the results in Figure 12 show that, at large distances, the drone position estimates of our proposed system are similar to those of GNSS in outdoor environments. This accuracy is enough to help the drone approach the platform. Furthermore, when we are near the platform, the accuracy of our system improves significantly, as we can see in Table 8, where the drone flew as far as 4.5 m from the platform. Thus, when the drone starts its landing operation, the accuracy of the system is good enough to help it land on the platform.

### 5.3. Comparison with State of the Art Technologies

Table 10 presents a comparison of the proposed system with others in the literature. This table shows the characteristics of a UWB-based LAS, such as the one proposed in [42], while the accuracy indicated is the one presented in Section 5.1.1. We can observe that this system achieved the worst accuracy of the compared LAS systems. The authors of [19] used vision in their LAS and presented a high accuracy in indoor environments and short ranges. However, this vision-based LAS was tested at a much lower horizontal velocity than our proposed LAS. Our proposal combines UWB technology with IMUs and magnetometers and, thus, achieves a high positioning rate, which is crucial for autonomous landing. Moreover, our proposed LAS is not affected by lighting conditions, as is the case with vision-based systems. We have shown that it can find the landing platform from at least 20 m and is robust in terms of high horizontal velocities and accelerations.

## 6. Conclusions and Future Research

This paper presents a novel landing assistance system capable of locating a UAV for a safe landing after its inspection mission. The proposed LAS is composed of eight UWB anchors placed around the landing platform of the drone and two UWB tags on the vehicle. Both tags also contain an IMU and a magnetometer, which enables the combination of real time acceleration of the drone with the UWB data. Unlike other proposed solutions in the literature, our LAS neither needs a large infrastructure deployment, nor does it depend on lighting conditions or the availability of GNSS.

In a recent study, a similar deployment was proposed for a UWB-based RTLS of an autonomous drone. In contrast to this study, our research tested several flights with different horizontal accelerations, so that the effect of sudden changes of the movement of the drone, which could be caused by windy weather, could be studied. It has been concluded that higher accelerations can cause problems in UWB-based RTLSs, as their positioning rate can be too low for correct tracking of the drone’s movements.

Our proposed LAS is more accurate than UWB-based systems when the drone suffers from high accelerations thanks to the fusion of UWB data with different sensors, namely IMUs and magnetometers. Our proposed algorithm takes advantage of the high sampling rate of the IMUs to estimate the position of the drone with a higher rate. Thus, it achieves a better tracking performance of the drone in those flights of high velocity and/or acceleration. Moreover, the proposed combination of tags’ positions further improves the accuracy of our LAS. Higher robustness is gained because possible errors from one of the tags are compensated with the other. As a result, with our novel LAS, an RMSE value of 0.208 m was obtained, compared to an RMSE value of 0.410 m of a traditional UWB-based LAS. Thanks to the higher accuracy and sampling rate of our proposal, the decision-making of an autonomous vehicle becomes safer.

Additionally, measurements in an outdoor relevant environment have shown that our system is able to position the drone when it is flying close to the landing platform and to track it accurately until the end of the flight. When the drone is flying far away from the landing platform our system presents an accuracy similar to GNSS. However, when the drone is near to the landing platform, our LAS presented better accuracy than GNSS. Furthermore, compared with vision-based systems in the literature, our LAS is not sensitive to lighting conditions. This will allow it to be used with a drone that inspects the inside of critical infrastructures such as off-shore wind turbines or a tank in a petrochemical plant.

In conclusion, this paper has presented an accurate landing assistance system for autonomous drones that combines UWB with IMU and magnetometer data. The system can also be improved to obtain a higher flexibility. For example, the case of a moving platform has not been considered, so future research lines could point towards this direction.

## Figures and Tables

**Table 1:** Configuration parameters of the UWB system.

Parameter	Value	Units
Carrier frequency	3.9936	GHz
Bandwidth	499.2	MHz
Channel	2	-
Bitrate	6.8	Mbps
PRF (pulse repetition frequency)	16	MHz
Preamble length	128	symbols
Preamble code	3	-
SFD (start of frame delimiter)	8	symbols
Ranging rate	3.3	Hz

**Table 2:** Configuration parameters of MotionFX.

Parameter	Value
Sampling rate	25 Hz
output_type	1
acc_orientation	ENU
gyro_orientation	ENU
mag_orientation	ESU
LMode	1
ATime	0.9
MTime	1.5
FrTime	0.667
modx	2

**Table 3:** Positions of the anchors during the tests.

Anchor Name	x (m)	y (m)	z (m)
A0	1.998	0.0	0.145
A1	1.0	0.0	0.149
A2	0.0	0.0	0.147
A3	0.0	0.999	0.151
A4	0.0	1.998	0.155
A5	1.001	1.998	0.153
A6	1.998	1.998	0.157
A7	1.998	0.999	0.159

**Table 4:** Measured horizontal acceleration on the centre of the drone.

Flight	Mean Acceleration ($m/s^{2}$)	Max Acceleration ($m/s^{2}$)
Flight 1	0.456	1.632
Flight 2	0.483	1.651
Flight 3	0.445	1.588
Flight 4	0.563	1.838
Flight 5	1.092	4.145
Flight 6	1.958	6.496
Flight 7	1.404	4.412
Flight 8	1.374	4.199
Flight 9	1.285	4.807

**Table 5:** Accuracy using only UWB data with four anchors.

Tag	Flight	*μ* (m)	*σ* (m)	RMSE (m)	P$(\epsilon_{p})\;<\;80\%$ (m)	P(%) < 1 m (%)	*ϵ*_*max*_ (m)
T1	Flight 1	0.233	0.144	0.273	0.346	100	0.608
	Flight 2	0.245	0.148	0.286	0.384	100	0.598
	Flight 3	0.214	0.176	0.277	0.336	100	0.751
	Flight 4	0.273	0.161	0.317	0.407	100	0.685
	Flight 5	0.264	0.279	0.383	0.356	97.46	1.609
	Flight 6	0.227	0.161	0.278	0.372	100	0.681
	Flight 7	0.311	0.249	0.398	0.490	97.67	1.075
	Flight 8	0.339	0.245	0.418	0.566	98.12	1.214
	Flight 9	0.411	0.323	0.522	0.618	94.95	1.709
	All	0.293	0.238	0.377	0.461	98.39	1.709
T2	Flight 1	0.184	0.133	0.227	0.318	100	0.565
	Flight 2	0.221	0.160	0.273	0.375	100	0.649
	Flight 3	0.256	0.233	0.346	0.455	100	0.854
	Flight 4	0.327	0.246	0.409	0.580	100	0.932
	Flight 5	0.336	0.346	0.481	0.529	93.78	1.716
	Flight 6	0.301	0.302	0.426	0.480	96.00	1.499
	Flight 7	0.369	0.397	0.541	0.666	92.35	1.790
	Flight 8	0.333	0.310	0.455	0.551	94.14	1.283
	Flight 9	0.433	0.387	0.580	0.736	90.15	2.001
	All	0.316	0.309	0.442	0.521	95.71	2.001

**Table 6:** Accuracy using only UWB data with eight anchors.

Tag	Flight	*μ* (m)	*σ* (m)	RMSE (m)	P$(\epsilon_{p})\;<\;80\%$ (m)	P(%) < 1 m (%)	*ϵ*_*max*_ (m)
T1	Flight 1	0.226	0.161	0.277	0.334	100	0.720
	Flight 2	0.237	0.116	0.263	0.356	100	0.528
	Flight 3	0.208	0.153	0.258	0.347	100	0.774
	Flight 4	0.240	0.135	0.275	0.365	100	0.581
	Flight 5	0.278	0.290	0.401	0.460	96.98	1.431
	Flight 6	0.259	0.205	0.330	0.498	100	0.865
	Flight 7	0.265	0.196	0.329	0.450	100	0.789
	Flight 8	0.343	0.238	0.417	0.571	99.38	1.067
	Flight 9	0.357	0.289	0.459	0.554	95.38	1.304
	All	0.280	0.222	0.357	0.445	98.86	1.431
T2	Flight 1	0.169	0.113	0.203	0.279	100	0.492
	Flight 2	0.204	0.144	0.250	0.323	100	0.650
	Flight 3	0.246	0.224	0.333	0.433	100	0.857
	Flight 4	0.275	0.206	0.344	0.469	100	0.904
	Flight 5	0.342	0.370	0.503	0.566	90.31	1.623
	Flight 6	0.379	0.360	0.522	0.737	91.71	1.722
	Flight 7	0.327	0.269	0.423	0.555	97.00	1.267
	Flight 8	0.324	0.268	0.420	0.582	99.33	1.099
	Flight 9	0.372	0.325	0.493	0.611	93.81	1.487
	All	0.303	0.282	0.414	0.505	96.69	1.722

**Table 7:** Accuracy data fusing UWB and inertial data.

Tag	Flight	*μ* (m)	*σ* (m)	RMSE (m)	P$(\epsilon_{p})\;<\;80\%$ (m)	P(%) < 1 m (%)	*ϵ*_*max*_ (m)
T1	Flight 1	0.175	0.105	0.204	0.265	100	0.630
	Flight 2	0.199	0.122	0.233	0.306	100	0.613
	Flight 3	0.176	0.094	0.200	0.245	100	0.578
	Flight 4	0.193	0.077	0.208	0.259	100	0.434
	Flight 5	0.165	0.103	0.194	0.241	100	0.751
	Flight 6	0.195	0.139	0.240	0.298	100	0.814
	Flight 7	0.193	0.151	0.245	0.302	99.82	1.090
	Flight 8	0.207	0.153	0.257	0.336	100	0.794
	Flight 9	0.243	0.179	0.301	0.380	99.70	1.203
	All	0.198	0.137	0.241	0.296	99.93	1.203
T2	Flight 1	0.177	0.118	0.213	0.289	100	0.496
	Flight 2	0.167	0.110	0.200	0.252	100	0.523
	Flight 3	0.155	0.114	0.192	0.262	100	0.577
	Flight 4	0.225	0.187	0.293	0.349	100	0.941
	Flight 5	0.209	0.135	0.249	0.326	100	0.791
	Flight 6	0.237	0.201	0.310	0.398	99.45	1.314
	Flight 7	0.218	0.137	0.258	0.346	100	0.731
	Flight 8	0.236	0.185	0.300	0.368	99.49	1.269
	Flight 9	0.223	0.156	0.272	0.325	99.79	1.060
	All	0.210	0.159	0.263	0.329	99.82	1.314

**Table 8:** Accuracy data when combining tags.

Flight	μ (m)	σ (m)	RMSE (m)	$P(\epsilon_{p})\;<\;80\%$ (m)	P(%) < 1 m (%)	$\epsilon_{\max}$ (m)
Flight 1	0.145	0.081	0.166	0.234	100	0.350
Flight 2	0.142	0.088	0.167	0.230	100	0.403
Flight 3	0.129	0.083	0.154	0.196	100	0.417
Flight 4	0.156	0.095	0.182	0.223	100	0.473
Flight 5	0.157	0.103	0.188	0.244	100	0.546
Flight 6	0.195	0.156	0.250	0.321	100	0.862
Flight 7	0.179	0.135	0.224	0.299	100	0.660
Flight 8	0.182	0.139	0.229	0.296	100	0.845
Flight 9	0.199	0.154	0.251	0.317	99.67	1.150
All	0.168	0.124	0.208	0.260	99.95	1.150

**Table 9:** Comparison of all cases.

System	*μ* (m)	*σ* (m)	RMSE (m)	P$(\epsilon_{p})\;<\;80\%$ (m)	P(%) < 1 m (%)	*ϵ*_*max*_ (m)
UWB as [42]	0.304	0.275	0.410	0.488	97.10	2.001
Proposal	0.168	0.124	0.208	0.260	99.95	1.150

**Table 10:** Comparison with state of art the systems.

	Indoor Accuracy RMSE		Maximum Tested Indoor Range	Maximum Indoor Horizontal Velocity	Maximum Tested Outdoor Range	Minimum Positioning Rate	Sensitivity to Lighting Conditions
	X (m)	Y (m)	(m)	(m/s)	(m)	(Hz)	
Vision [19]	0.012	0.014	2	0.190	-	10	Yes
UWB as [42]	0.355	0.205	4.5	3.139	-	3.3	No
This work	0.177	0.115	4.5	3.139	20	25	No

## Data Availability

Not applicable.

## Abbreviations

AGV	Automated Guided Vehicle
EKF	Extended Kalman Filter
FMCW	Frequency Modulated Continuous Wave
GNSS	Global Navigation Satellite System
IMU	Inertial Measurement Unit
IR-UWB	Impulse Radio Ultra-wideband
LAS	Landing Assistance System
LIDAR	Laser Imaging Detection and Ranging
PRF	Pulse Repetition Frequency
RLS	Recursive Least Squares
RMSE	Root Mean Square Error
RTK-GPS	Real-time Kinematic Global Positioning System
RTLS	Real Time Locating System
SBC	Single Board Computer
SFD	Start of Frame Delimiter
ToF	Time-of-Flight
TWR	Two-Way Ranging
UAV	Unmanned Aerial Vehicle
UWB	Ultra-wideband
